# Spray-Dried Chia Oil Oleogel Microcapsules from Pickering Emulsions: Wall Material Effects on Encapsulation Efficiency and In Vitro Lipid Digestion

**DOI:** 10.3390/gels12090856

**Published:** 2026-09-20

**Authors:** Eduardo Morales, Matias Leiva, Mónica Rubilar, Marcela Quilaqueo, Sonia Millao, César Burgos-Díaz, Ever Hernández-Olivas

**Affiliations:** 1 Scientific and Technological Bioresource Nucleus (BIOREN), Universidad de La Frontera, Avenida Francisco Salazar 01145, Temuco 4811230, Chile; monica.rubilar@ufrontera.cl (M.R.); marcela.quilaqueo@ufrontera.cl (M.Q.); sonia.millao@ufrontera.cl (S.M.); 2 Department of Chemical Engineering, Faculty of Engineering and Science, Universidad de La Frontera, Temuco 4811230, Chile; m.leiva04@ufromail.cl; 3 Agriaquaculture Nutritional Genomic Center (CGNA), Temuco 4781158, Chile; cesar.burgos@cgna.cl; 4 InnograinLab, Food Technology Division, Department of Agricultural Engineering, University of Valladolid, 47011 Valladolid, Spain; everhdzo@gmail.com; 5 Institute of Sustainable Processes, University of Valladolid, 47011 Valladolid, Spain

**Keywords:** chia oil oleogels, lupin protein aggregates, Pickering emulsions, spray drying, lipid digestion

## Abstract

The objective of this study was to develop chia oil oleogel microcapsules by spray-drying Pickering emulsions (OLG/W–E) stabilized with lupin protein aggregates (LPA), optimize spray-drying conditions, and evaluate the effect of wall composition on encapsulation efficiency (EE) and in vitro lipid digestion. Oleogels structured with beeswax (BW) and shellac wax (SW) at 2.5 and 5.0% (*w*/*w*) were characterized for oil-binding capacity (OBC) and firmness. OLG/W–E were formulated, and spray drying was optimized using a Taguchi design considering inlet air temperature (120 and 160 °C), emulsion type (OLG_SW_/W–E and OLG_BW_/W–E), and wall material (maltodextrin, MD, and gum arabic, GA). Increasing wax concentration to 5% enhanced OBC and firmness, while 3% LPA provided adequate stability. The optimal condition was achieved at 160 °C using a BW-structured oleogel and GA, yielding an EE of 71.3 ± 1.25%. Under optimized conditions, replacing GA with up to 1% (*w*/*w*) sodium alginate (SA) did not significantly affect moisture content, solubility, process yield, or EE (*p* > 0.05). However, SA significantly increased lipid digestion (*p* < 0.05), with FFA release increasing from 26.4 ± 3.23% for GA microcapsules to 44.8 ± 6.33% and 43.4 ± 0.30% for GA/SA–0.5% and GA/SA–1.0%, respectively. These results indicate that SA can modulate lipid digestion during gastrointestinal digestion while maintaining comparable physicochemical properties.

## 1. Introduction

Vegetable oils rich in polyunsaturated fatty acids (PUFAs) play a crucial role in promoting human health and well-being by reducing the risk of cardiovascular and neurodegenerative diseases, arthritis, diabetes, and certain cancers [1]. In this context, chia oil stands out for its high PUFA content (84.1–85.0%), mainly omega-3 fatty acids (64.5–69.3%), and also contains bioactive compounds such as tocopherols, polyphenols, carotenoids, and phosphorus [2]. However, PUFA-rich oils are more susceptible to lipid oxidation than saturated oils (solid vegetable fats), which can lead to the formation of compounds responsible for undesirable sensory characteristics and, consequently, negatively affect food quality [3].

In recent years, structured vegetable oils, also known as oleogels, have attracted increasing attention from the scientific community, particularly among researchers focused on developing lipid-based ingredients [4]. Oleogelation, the process of structuring liquid oils, involves immobilizing the oil within a thermoreversible three-dimensional network formed by different oleogelators, such as natural waxes, resulting in materials with viscoelastic properties comparable to those of conventional fats [5]. These systems can exhibit a solid or semisolid texture, high firmness, excellent oil-binding capacity, and enhanced resistance to lipid oxidation, as demonstrated for vegetable oil oleogels structured with beeswax (BW) [6,7,8] and shellac wax (SW) [9,10,11]. Despite these advantages, the use of oleogels as healthy food ingredients remains limited due to their poor compatibility with liquid and powdered food systems. To overcome this limitation, oleogels have increasingly been incorporated as the dispersed oil phase in oil-in-water (O/W) emulsions, giving rise to oleogel-in-water (OLG/W) emulsions. This strategy not only broadens the technological applications of oleogels but also improves oil retention, oxidative stability, and shelf life by forming a crystalline network within the dispersed oil droplets [12,13,14].

Among the different approaches developed to stabilize OLG/W systems, Pickering emulsions have emerged as one of the most promising alternatives because they provide superior physical stability compared with conventional surfactant-stabilized emulsions. In recent years, increasing attention has therefore been devoted to developing Pickering OLG/W emulsions stabilized by food-grade particles [13,15]. Unlike conventional emulsions stabilized by molecular surfactants, Pickering emulsions rely on the irreversible adsorption of solid colloidal particles at the oil–water interface, thereby forming a robust interfacial barrier that effectively prevents droplet coalescence and enhances emulsion stability [16]. Consequently, the selection of suitable food-grade particles plays a critical role in determining the physicochemical stability and functionality of these systems. To date, several studies have successfully stabilized OLG/W Pickering emulsions using colloidal particles derived from whey protein aggregates [17,18], citrus fiber–whey protein isolate complexes [19], and pea protein [13].

Despite recent advances in the development of stable OLG/W Pickering emulsions, little attention has been paid to preserving their structural integrity during dehydration. Spray drying is the most widely adopted technique for producing powdered emulsions because it improves the shelf life of unsaturated oils by limiting lipid oxidation, reducing microbial growth, and simplifying storage and transportation [20,21]. However, the thermal and mechanical stresses generated during spray drying may alter the interfacial structure of emulsions, resulting in destabilization, droplet disruption, and oil leakage [22]. Therefore, obtaining highly stable OLG/W Pickering emulsions and selecting appropriate wall materials prior to spray drying are key factors for producing powders with high encapsulation efficiency [22]. Although spray-dried Pickering systems containing structured lipid phases have been increasingly investigated, the combined use of wax-structured chia oil, lupine protein aggregates as Pickering stabilizers, and polysaccharide wall matrices, particularly with respect to both encapsulation efficiency and lipid hydrolysis during gastrointestinal digestion, remains underexplored.

Given the critical role of wall materials in maintaining emulsion stability during drying and protecting encapsulated lipids during storage, considerable attention has been given to polysaccharide-based agents such as maltodextrin (MD), gum arabic (GA), and sodium alginate (SA). These materials are widely used in spray drying for their functional properties and suitability for food applications, although each has distinct advantages and limitations. MD, obtained by partial hydrolysis of starch, is valued for its high water solubility, low viscosity at high solids concentrations, and ability to protect lipids against oxidation; however, its poor emulsifying capacity limits its use as a sole wall material [23,24]. GA, a naturally occurring anionic polysaccharide–protein complex derived from Acacia species, offers excellent emulsifying properties and can enhance the stability of spray-dried oil microcapsules. However, its relatively high cost may limit large-scale industrial applications [15]. In contrast, SA has attracted increasing attention for its stability under acidic conditions and its ability to form gel networks in the presence of divalent cations, which can modulate lipid release during intestinal digestion. However, its high viscosity may hinder emulsion atomization, limiting its use as the sole wall material in spray-drying processes [25,26].

Based on the above, the present study investigated the encapsulation of chia oil oleogels using Pickering emulsions (OLG/W–E) combined with polysaccharide-based wall materials prior to spray drying. We hypothesized that integrating a structured lipid phase with a Pickering interface and a polysaccharide-based wall matrix could provide complementary control over oil retention during spray drying and lipid hydrolysis during gastrointestinal digestion. The objective of this study was to develop chia oil oleogel microcapsules by spray drying OLG/W–E stabilized with lupin protein aggregates (LPA) and to evaluate the effects of wall material composition on encapsulation efficiency (EE) and in vitro gastrointestinal digestion. Chia oil oleogels were first prepared using BW and SW at concentrations of 2.5 and 5.0% (*w*/*w*), and those exhibiting suitable semisolid properties were selected as the structured lipid phase for incorporation into LPA-stabilized OLG/W–E. The effect of LPA concentration on emulsion stability was assessed based on the creaming index. The resulting emulsions were then spray-dried, and a Taguchi experimental design was used to optimize the formulation by evaluating the effects of inlet air temperature (120 and 160 °C), emulsion type (OLG_SW_/W–E and OLG_BW_/W–E), and wall material type (MD and GA) on EE. Finally, SA was incorporated into the optimized wall material formulation at low concentrations to evaluate its effect on in vitro gastrointestinal digestion. This study provides new insights into the production of spray-dried OLG/W Pickering emulsions as functional powdered lipid ingredients, with potential applications in developing healthier food formulations.

## 2. Results and Discussion

### 2.1. Chia Oil Oleogel-in-Water (OLG/W) Pickering Emulsions

#### 2.1.1. Formulation and Characterization of Chia Oil Oleogel

**Figure 1:**
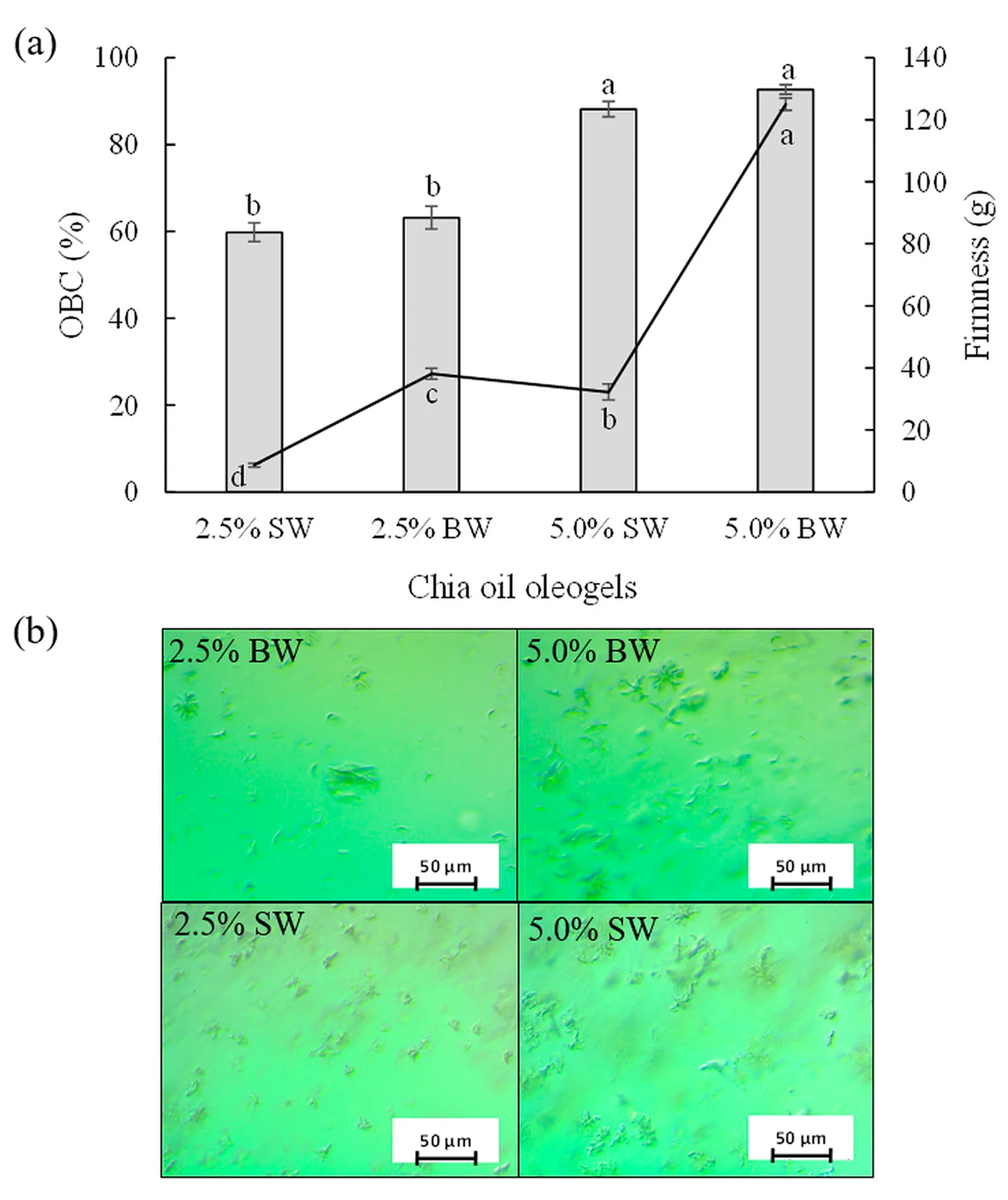
(**a**) Oil-binding capacity (OBC) and firmness, and (**b**) optical microscopy images of chia oil oleogels prepared with shellac wax (SW) and beeswax (BW) at concentrations of 2.5 and 5.0% (*w*/*w*). Different lowercase letters indicate significant differences among oleogel formulations (*p* < 0.05).

The oil binding capacity (OBC) results are presented in Figure 1. OBC is a key indicator of oleogel performance, reflecting the ability of the oleogelator network to entrap and retain liquid oil within the gel matrix [27].

Figure 1a shows the effect of shellac wax (SW) and beeswax (BW) concentrations on the OBC of chia oil oleogels. Samples formulated with 2.5% SW and BW exhibited OBC values of 59.8 ± 2.15% and 63.2 ± 2.62%, respectively, corresponding to oil losses of 40.2% and 36.8%. Increasing the oleogelator concentration to 5.0% (*w*/*w*) significantly enhanced (*p* < 0.05) OBC, reaching 88.2 ± 1.76% for SW and 92.7 ± 1.11% for BW, while oil loss decreased to 11.8% and 7.3%, respectively. This improvement is consistent with the formation of a denser and more interconnected crystalline network at higher wax concentrations, which promotes more efficient physical entrapment of liquid oil within the oleogel matrix [27,28]. No significant differences (*p* > 0.05) were observed between the two waxes at the same concentration, indicating that oleogelator concentration had a greater influence on OBC than wax type. These findings are consistent with those reported by Morales et al. [29], who demonstrated that increasing the concentration of natural wax-based oleogelators increases the solid fraction of chia oil oleogels and restricts oil mobility within the crystalline network. Although OBC improved at 5.0% wax concentration, this parameter alone was not considered sufficient to determine the suitability of the oleogels for subsequent emulsion preparation. Since the oleogels were melted before emulsification, firmness in the structured state was not considered a direct limitation to their dispersion during homogenization.

Figure 1a also presents the firmness of the chia oil oleogels. Unlike OBC, firmness was significantly influenced by both wax type and concentration. The oleogel formulated with 2.5% (*w*/*w*) SW exhibited the lowest firmness (8.7 ± 0.61 g), whereas increasing SW concentration to 5.0% significantly (*p* < 0.05) increased firmness to 32.3 ± 2.54 g. BW-based oleogels were significantly (*p* < 0.05) firmer than their SW counterparts at both concentrations, reaching 38.2 ± 1.76 g and 125 ± 2.0 g at 2.5% and 5.0% (*w*/*w*), respectively. The higher firmness of BW-based oleogels may be related to differences in crystal morphology and network connectivity that have been previously reported for natural waxes [30]. In general, increasing the oleogelator concentration increases the number of crystal junctions and solid fraction, reinforcing the crystalline network and increasing resistance to deformation [9,29]. This effect was reflected in the higher firmness observed at 5.0% oleogelator concentration. However, because the oleogels were melted before emulsification in the present study, the higher firmness of the 5.0% formulations did not represent an operational limitation during homogenization. The wax-based oleogels used in this study are formed by a crystalline network reported to be thermoreversible [5]. Thus, melting the oleogels before emulsification allowed the lipid phase to be processed in the liquid state, while subsequent cooling may promote the reformation of the crystalline structure. However, the extent of structural recovery after emulsification was not specifically evaluated in the present study.

To complement the quantitative evaluation of OBC and firmness, optical microscopy was used as a qualitative approach to visualize the crystalline organization of the oleogels (Figure 1b). Both SW- and BW-based oleogels exhibited increased crystal density with increasing oleogelator concentration. Oleogels prepared with 2.5% (*w*/*w*) wax displayed a more dispersed crystalline network, consistent with their lower OBC and firmness values. In contrast, the 5.0% formulations exhibited a denser and more interconnected structure, consistent with their higher values. No noticeable differences in the overall crystal distribution between SW- and BW-based oleogels were observed by optical microscopy. Thus, microscopy was considered complementary structural evidence, while OBC and firmness provided quantitative indicators of oil retention and mechanical properties, respectively. Because the oleogels are thermoreversible, they were melted prior to emulsification. Therefore, the 5.0% SW and BW oleogels were selected for subsequent emulsion preparation based primarily on their superior OBC. Previous studies have demonstrated that stable OLG/W emulsions can be obtained using oleogels structured with low concentrations of natural waxes and stabilized with Pickering particles based on protein aggregates [13,18] or protein/polysaccharide complexes [22]. Accordingly, the effect of the concentration of plant protein-derived Pickering particles on the physical stability and microstructure of OLG/W emulsions prepared using chia oil oleogels was subsequently investigated.

#### 2.1.2. Formulation and Characterization of Chia Oil Oleogel-in-Water (OLG/W) Pickering Emulsions

The results obtained in this study indicate that lupin protein aggregates (LPA) are also effective stabilizers for chia oil oleogel-in-water (OLG/W) Pickering emulsions. These findings broaden previous evidence demonstrating the ability of LPA to stabilize conventional oil-in-water (O/W) Pickering emulsions while preserving emulsion stability during spray drying, thereby enhancing the retention of bioactive compounds such as astaxanthin [31]. However, their use as Pickering stabilizers for OLG/W emulsions has not been reported previously.

**Figure 2:**
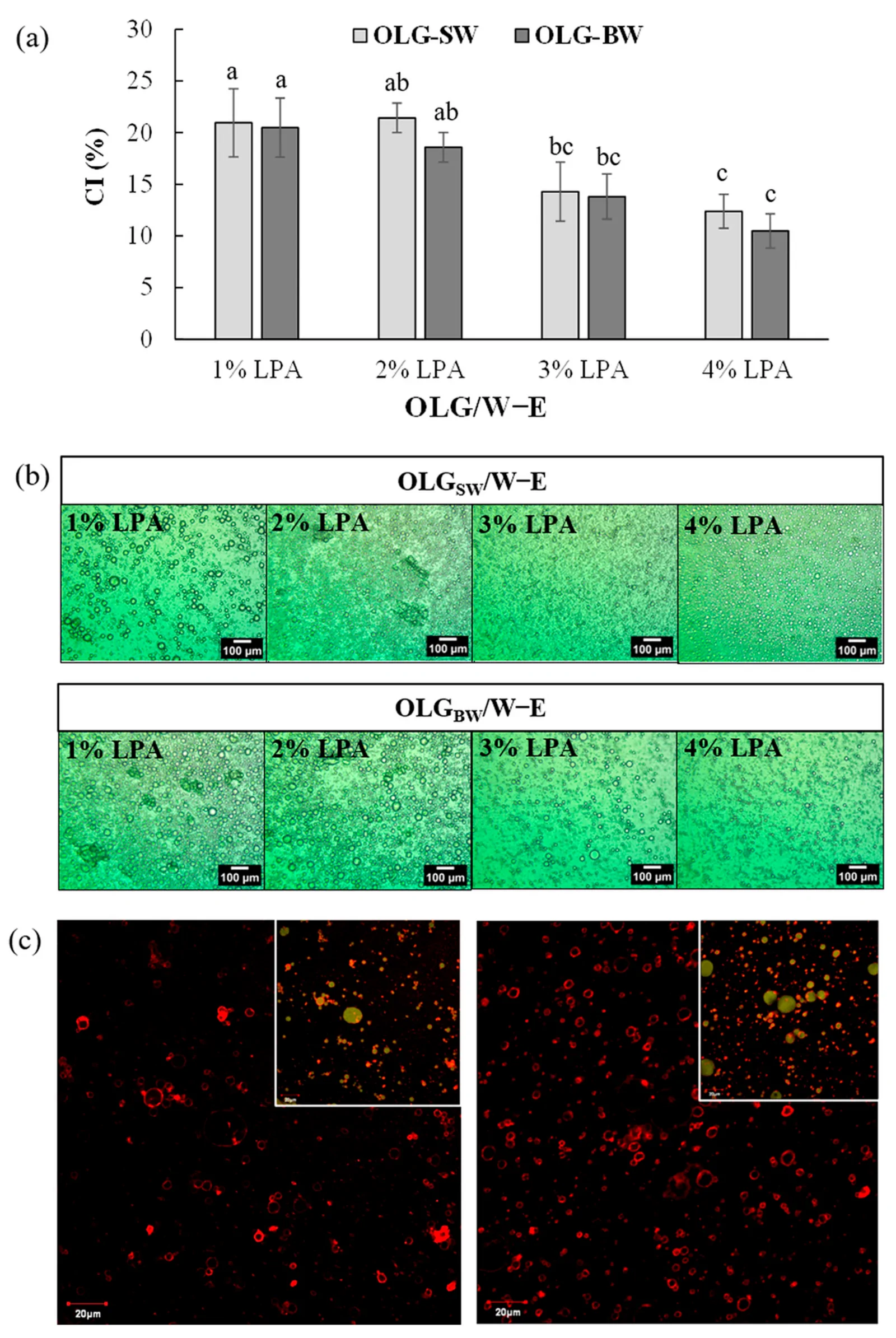
(**a**) Creaming index (CI, %) and (**b**) light microscopy images of Pickering emulsions (OLG_SW_/W–E and OLG_BW_/W–E) stabilized with 1–4% (*w*/*w*) LPA. (**c**) CLSM images of Pickering emulsions stabilized with 3% (*w*/*w*) LPA. The protein aggregates were stained with Rhodamine B (red), revealing their localization at the oleogel–water interface, while the structured chia oil phase was stained with Nile red, showing greenish fluorescence within the droplets. Different letters indicate statistical differences between emulsions (*p* < 0.05).

Figure 2 shows the effect of increasing concentrations of lupin protein aggregates (LPA), used as Pickering stabilizers, on the physical stability of OLG/W emulsions prepared with chia oil oleogels structured with SW or BW at 5.0% (*w*/*w*). To further verify the formation of LPA at the selected concentration, the 3% (*w*/*v*) lupin protein dispersion was examined by optical microscopy before and after thermal treatment. While no clearly distinguishable aggregates were observed in the untreated dispersion, the thermally treated and cooled dispersion showed visible protein aggregates (Figure S1, Supplementary Materials), supporting the formation of LPA under the conditions used in this study. Physical stability was evaluated using the creaming index (CI), which reflects the gravitational separation resulting from the density difference between the dispersed and continuous phases [14]. The CI was determined after 24 h of storage for emulsions formulated with LPA concentrations ranging from 1 to 4% (*w*/*w*) (Figure 2a). As expected, the physical stability of the emulsions improved progressively with increasing Pickering particle concentration, as evidenced by a significant reduction (*p* < 0.05) in CI. Emulsions prepared with SW- and BW-structured oleogels and stabilized with 1% (*w*/*w*) LPA exhibited similar CI values of 21.0 ± 3.30 and 20.5 ± 2.86%, respectively. Increasing the LPA concentration to 2% (*w*/*w*) resulted in a slight decrease in CI; however, no significant differences (*p* > 0.05) were observed between the two emulsion systems. In contrast, increasing the LPA concentration to 3% (*w*/*w*) significantly reduced (*p* < 0.05) the CI to 14.3 ± 2.86 and 13.8 ± 2.18% for emulsions prepared with SW- and BW-based oleogels, respectively. No significant differences (*p* > 0.05) were detected between emulsions formulated with 3 and 4% (*w*/*w*) LPA, regardless of oleogel type, indicating that increasing the LPA concentration above 3% did not further reduce creaming under the conditions evaluated. Therefore, 3% (*w*/*w*) LPA was selected as the minimum concentration providing sufficient physical stability for subsequent spray drying. This enhanced stability can be attributed to the more efficient adsorption of protein aggregates at the oil–water interface, where they form a continuous protective layer around the droplets, creating a physical barrier that suppresses droplet migration and creaming [32]. In addition, Wei et al. [13] reported that some vegetable protein aggregates acting as Pickering particles remain dispersed in the aqueous phase and interact to form a weak three-dimensional network. The resulting network increases the viscosity of the continuous phase and restricts droplet mobility, thereby reducing creaming and other destabilization mechanisms.

To further assess whether this stabilization behavior was influenced by oil structuring, Pickering O/W emulsions containing unstructured chia oil were also prepared as controls under the same conditions. Their CI values followed a trend similar to those of emulsions containing structured chia oil, suggesting comparable physical stability between the two systems under the conditions evaluated. In both cases, 3% (*w*/*w*) LPA was sufficient to achieve adequate emulsion stability. These control results are provided in Supplementary Figure S2.

The optical microscopy images in Figure 2b qualitatively support the reduction in CI observed as LPA concentration increases. As the concentration of Pickering particles increased, the emulsions appeared to exhibit smaller droplets and a more homogeneous droplet distribution. In contrast, emulsions formulated with 1 and 2% (*w*/*w*) LPA showed visually larger droplets and a broader size distribution, suggesting less effective interfacial stabilization. Emulsions stabilized with 3 and 4% (*w*/*w*) LPA displayed a more homogeneous microstructure, consistent with the lower CI values observed in Figure 2a. The equivalent diameter (Deq) distribution of both types of Pickering OLG/W emulsions stabilized with 3% LPA showed a single predominant droplet population, with the highest frequency observed in the 5–15 μm range. This distribution was supported by the optical micrographs presented in Figure 2b, which showed a gradual decrease in droplet size and a more homogeneous distribution as the LPA concentration increased (Figure S3, Supplementary Materials). These findings are consistent with recent studies on OLG/W Pickering emulsions stabilized by protein aggregates, which have demonstrated that increasing particle concentration promotes more complete interfacial coverage, reduces droplet size, and significantly improves the physical stability of the emulsions [13,14].

Therefore, the CI and optical microscopy results demonstrated a progressive improvement in the physical stability of the OLG/W emulsions as the LPA concentration increased. The reduction in CI was accompanied by a decrease in droplet size and the development of a more homogeneous microstructure, indicating that a minimum LPA concentration of 3% (*w*/*w*) was sufficient to stabilize the OLG/W Pickering emulsions prior to spray drying effectively. To gain further insight into the stabilization mechanism of the OLG/W Pickering emulsions, the interfacial microstructure was examined by confocal laser scanning microscopy (CLSM).

Figure 2c showed a red fluorescent signal surrounding the droplets of both Pickering emulsions (OLG_SW_/W–E and OLG_BW_/W–E), consistent with the localization of the protein aggregates at the oleogel–water interface. The structured chia oil phase was also visualized by Nile red, showing greenish fluorescence within the droplets (Figure 2c, upper-right insets). No noticeable differences were observed between emulsions prepared with SW- and BW-based oleogels, as both exhibited similar fluorescence patterns. These observations support the interfacial localization of the lupin protein aggregates and their role as Pickering stabilizers. Together with the CI and optical microscopy results, the CLSM observations supported the selection of 3% (*w*/*w*) LPA for preparing OLG/W Pickering emulsions prior to spray drying. These observations are consistent with the findings of Wei et al. [13], who demonstrated that thermally induced plant protein aggregates can adsorb almost irreversibly at the oil–water interface and form a dense protective layer surrounding emulsion droplets. Likewise, previous studies have reported that a high degree of interfacial coverage indicates that protein aggregates act as effective Pickering stabilizers by creating a physical barrier that prevents direct droplet contact and reduces coalescence [32,33]. Finally, the CLSM observations corroborated the results obtained from the creaming index and optical microscopy analyses, confirming that an LPA concentration of 3% (*w*/*w*) provided continuous and sufficient interfacial coverage to produce stable OLG/W Pickering emulsions with a well-defined microstructure suitable for subsequent spray drying.

### 2.2. Encapsulation of Oleogel-in-Water Pickering Emulsions by Spray Drying

#### 2.2.1. Formulation and Characterization of Chia Oil Oleogel Microcapsules

Spray drying was applied to transform OLG_SW_/W and OLG_BW_/W Pickering emulsions into powdered ingredients. The drying parameters were optimized using the Taguchi experimental design (Section 4.5). The effects of three independent variables, namely dryer inlet air temperature (120 and 160 °C), emulsion type (OLG_SW_/W–E and OLG_BW_/W–E), and wall material (MD and GA), were investigated to determine their influence on EE. The EE values obtained ranged from 55.3 to 70.5%. EE is a key indicator of microencapsulation performance, as it reflects the proportion of the active compound retained within the encapsulating matrix. Higher EE values indicate greater entrapment and protection of the bioactive compound inside the microcapsules [34].

**Figure 3:**
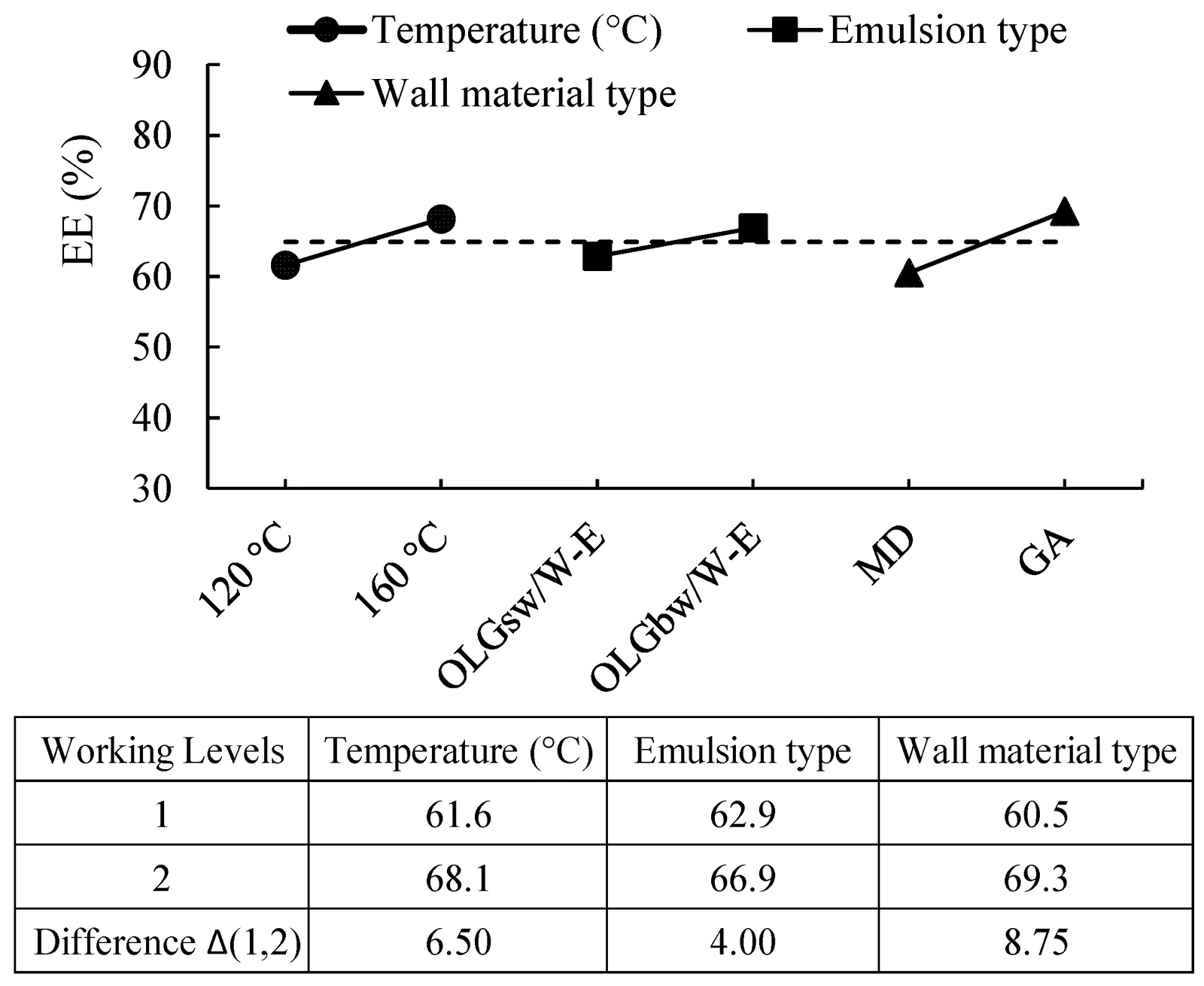
Main effects of the evaluated factors on encapsulation efficiency (EE), calculated from the mean EE at each factor level.

Figure 3 shows the effect of the working levels of each variable on EE. The effect of each variable was calculated as the absolute difference between the mean EE values obtained at its two evaluated levels. Thus, for each factor, the mean EE at one level was compared with that obtained at the other level, and the resulting difference represented the magnitude of its effect on EE. The results showed that the wall material, corresponding to GA, was the variable with the greatest influence on EE, with a difference of 8.75 units between the evaluated levels. The superior performance of GA compared with MD may be related to its film-forming properties and its ability to contribute to the formation of a protective matrix during spray drying [35]. This film enhances emulsion stability and limits oil migration toward the particle surface, thereby promoting greater retention of the lipid phase during drying [16]. In contrast, MD primarily acts as a matrix-forming agent and, due to its limited interfacial activity, generally requires combination with other biopolymers to provide more effective protection of the oil during spray drying [36].

The second most influential factor affecting EE was the spray-dryer inlet air temperature, with a difference of 6.50 units between the evaluated levels. Increasing the inlet temperature from 120 to 160 °C resulted in higher EE because the rapid evaporation of water promoted the early formation of a solid crust on the droplet surface. This barrier restricted oil migration toward the particle surface during drying, thereby reducing surface oil content and improving EE [34]. Previous studies have shown that insufficient inlet temperatures reduce the water evaporation rate, resulting in powders with higher moisture content, lower flowability, and a greater tendency to agglomerate. Conversely, excessively high inlet temperatures accelerate water evaporation, which may induce cracks in the wall material and promote the release, degradation, or loss of the encapsulated oil [20,21]. These findings indicate that an inlet temperature of 160 °C provided suitable drying conditions to maximize EE.

Among the variables evaluated, the emulsion type prepared with chia oil oleogels structured using SW and BW showed the smallest contribution to EE, with a difference of 4.0 units between the analyzed levels. However, the microcapsules obtained from OLG_BW_/W–E exhibited slightly higher EE than those produced from OLG_SW_/W–E. These results suggest that structuring chia oil into BW-based oleogels more effectively restricted lipid mobility than SW-based oleogels, which is consistent with the higher OBC previously observed for these formulations. As a result, oil migration toward the surface of the microcapsules produced from BW-based oleogel emulsions was reduced during spray drying, leading to lower surface oil content and, ultimately, improved EE. A similar trend was reported by [22], who obtained higher EE in spray-dried edible oil powders produced from OLG/W Pickering emulsions stabilized with soy protein/cellulose nanofibrils. In their study, incorporating 2% BW as the oil-structuring agent significantly improved EE compared with conventional Pickering O/W emulsions.

These observations were further supported by the ANOVA, which quantified the relative contribution of each independent variable to EE. ANOVA showed that wall material was the main contributor to the variation in EE (55.8%), followed by inlet air temperature (30.8%) and emulsion type (11.7%), confirming the dominant role of the wall material in maximizing EE. Based on the optimization analysis, the combination of a dryer air inlet temperature of 160 °C, an OLG_BW_/W–E, and GA as the wall material provided the highest EE. Furthermore, the optimized theoretical equation (OTE) predicted an EE of 74.5%, which closely matched the experimental value of 71.3 ± 1.25% obtained under the optimal processing conditions. The experimental EE was reasonably close to the value predicted by the Taguchi model, providing experimental support for the selected factor combination under the conditions evaluated.

On the other hand, to evaluate the effect of structuring chia oil as an oleogel on EE, the microcapsules obtained under the optimal conditions were compared with control microcapsules prepared from an O/W Pickering emulsion containing unstructured chia oil, while maintaining the same drying conditions and using GA as the wall material. The control microcapsules exhibited an EE of 68.5 ± 1.80%, which was slightly lower than that of the optimal formulation (71.3 ± 1.25%); however, no significant difference was observed between the two formulations (*p* > 0.05). Thus, the numerical difference in EE suggests that oil structuring with BW had a limited effect on EE under the conditions evaluated. These results do not provide sufficient evidence to conclusively establish that oil structuring enhances retention of the lipid phase during spray drying. The observed trend may instead be associated with the lower mobility of the structured oil, which could reduce its migration toward the microcapsule surface.

To determine whether the improvement in EE achieved under the optimized processing conditions also resulted in greater oxidative protection of the encapsulated oil, the accelerated oxidative stability of the samples corresponding to the experimental design, the optimal formulation, and the control prepared with unstructured chia oil was evaluated. The accelerated oxidative stability was evaluated in terms of the induction period (IP), which corresponds to the time required for a rapid increase in the lipid oxidation rate [37]. No significant differences (*p* > 0.05) were observed among the evaluated samples, with IP values ranging from 3.40 to 3.46 h (Table S1, Supplementary Materials). These results indicate that maximizing EE through process optimization did not necessarily translate into greater oxidative protection of the structured chia oil compared with the control. Although the optimized formulation exhibited greater retention of the lipid phase within the encapsulating matrix, this difference was not sufficient to produce detectable changes in oxidative stability under the conditions evaluated. The absence of significant differences may be related to the limited thermal exposure time during spray drying [20,21]. Although the process exposes Pickering emulsions to elevated temperatures, which could promote oxidation of highly unsaturated oils such as chia oil, the thermal exposure time during spray drying is typically limited to a few seconds. This short exposure period may favor the rapid formation of the encapsulating matrix and reduce the extent of thermal oxidation during processing [38]. However, the present experiments did not directly determine the contribution of residence time, matrix formation, or oxygen diffusion to the observed oxidative stability.

Following the identification of GA as the optimal wall material for maximizing the EE of chia oil oleogel microcapsules, the optimized GA-based formulation was further characterized for its physicochemical properties and morphology. To further investigate the effect of wall material composition, this formulation was used as the reference for a second formulation stage, in which GA was partially replaced with sodium alginate (SA) at proportions of 0.5 and 1.0% (*w*/*w*), while maintaining the total wall material concentration at 15% (*w*/*w*) (Table 1). Preliminary results indicated that SA incorporation should be limited to 0–1% (*w*/*w*) in the GA/SA mixture as a wall material in OLG/W Pickering emulsions, resulting in viscosity values ranging from 50 ± 1.41 to 121 ± 2.83 cP. Increasing the SA proportion to 1.5% (*w*/*w*) significantly increased (*p* < 0.05) the emulsion viscosity to 200 ± 4.24 cP, thereby hindering atomization and adversely affecting the operational performance of the spray-drying process. According to the manufacturer’s specifications, high feed-solution viscosity can impair atomization, alter droplet formation, and compromise drying uniformity (Table S2, Supplementary Materials). Similar behavior was reported by Singh et al. [25], who developed chitosan-stabilized Pickering O/W emulsions using a mixture of maltodextrin and low concentrations of alginate (<1%) as the wall material. Under these conditions, the emulsions exhibited adequate performance during spray drying, while the incorporation of alginate improved the lipid release profile during in vitro digestion.

The selected GA/SA formulations were subsequently processed under the same optimal spray-drying conditions established by the experimental design, thereby enabling evaluation of the effect of wall material composition while keeping the processing conditions constant. The resulting microcapsules were then compared for EE, moisture content, solubility, powder yield, and morphological characteristics to assess whether partial replacement of GA with SA altered their physicochemical and structural properties and, consequently, influenced their behavior during in vitro gastrointestinal digestion.

The physicochemical characteristics of the chia oil oleogel microcapsules obtained by spray drying are presented in Table 1. The moisture content of the formulations prepared with GA (optimal) and the GA/SA–0.5% and GA/SA–1.0% mixtures ranged from 4.08 ± 0.16 to 4.71 ± 0.23%. A slight increase was observed with the incorporation of SA; however, no significant differences were detected among the formulations (*p* > 0.05), indicating that the partial replacement of GA with SA at the concentrations evaluated did not substantially affect water removal during drying. Similar results were reported by Singh et al. [25], who obtained a moisture content of 4.86 ± 0.33% when spray-drying Pickering O/W emulsions using maltodextrin and sodium alginate mixtures as wall materials. Regarding powder solubility, all formulations showed relatively high values, with a slight increase for the microcapsules prepared with the GA/SA mixture as wall material. The incorporation of SA may have contributed to this trend due to the hydrophilic nature of this polysaccharide; however, the magnitude of the change was insufficient to produce significant differences (*p* > 0.05). A similar behavior was reported by Burgos-Díaz et al. [31], who reported solubility values ranging from 72 to 84% (*w*/*w*) for astaxanthin microcapsules obtained from Pickering O/W emulsions using maltodextrin as the wall material and subsequently spray-dried.

The process yield values were relatively close, ranging from 35.2 ± 1.38 to 37.9 ± 1.44%, with no clear trend among the formulations, suggesting that the partial replacement of GA with SA did not consistently affect powder recovery. Since all formulations were processed under the same drying conditions and at a constant total wall material concentration, the small differences observed may be primarily attributed to inherent variations in powder recovery during spray drying. Comparable values have been reported for related spray-dried Pickering systems; for example, Singh et al. [25], obtained a process yield of 40.7 ± 1.35% when spray-drying Pickering O/W emulsions stabilized with sodium alginate–maltodextrin mixtures. In these powdered microencapsulated systems, the presence of oil in O/W emulsions may promote material deposition and accumulation in the drying chamber due to aggregate formation during atomization, thereby reducing the amount of powder recovered in the cyclone and, consequently, the overall process yield [20,21]. Although the powder recovery in the present study was relatively low, it was comparable to that reported for related spray-dried Pickering systems, indicating that the observed yields are within the range previously reported for similar formulations. Nevertheless, the relatively low powder recovery represents a limitation for process scale-up and should be considered when assessing the practical applicability of the optimized process.

**Figure 4:**
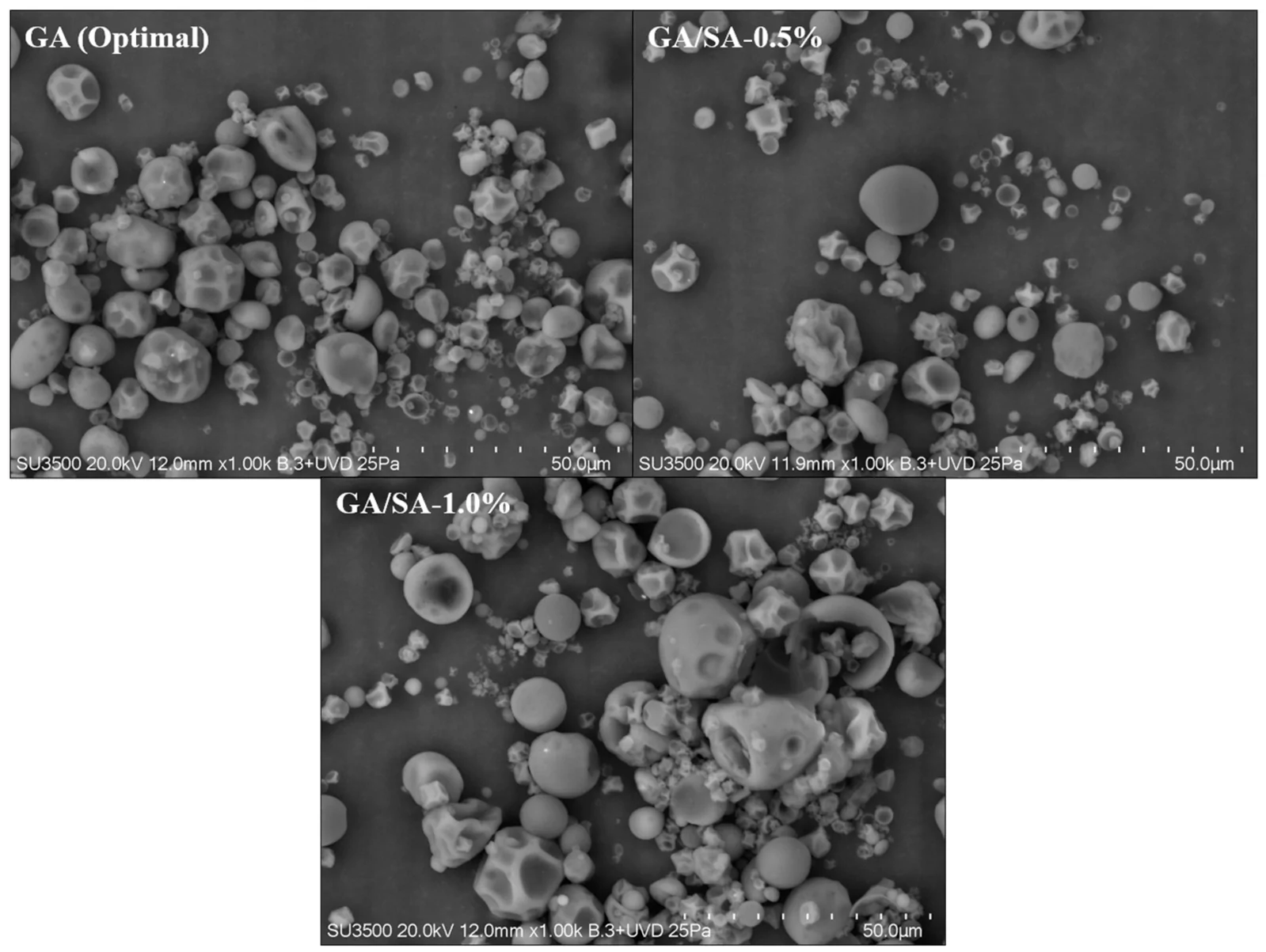
SEM images of chia oil oleogel microcapsules prepared with the optimized GA formulation and with GA/SA–0.5% and GA/SA–1.0% modified wall formulation.

The morphology of the chia oil oleogel microcapsules obtained using GA, GA/SA–0.5%, and GA/SA–1.0% as wall materials was examined by SEM (Figure 4). All three formulations exhibited predominantly spherical particles, along with some particles displaying irregular morphologies, surface concavities, and dents. No evident signs of agglomeration were observed, and the particles appeared to be predominantly smaller than 50 μm based on the scale bars of the SEM images. The presence of surface concavities has been attributed to rapid water evaporation and the consequent contraction of particles during spray-drying [25]. Overall, no evident morphological differences were observed among the formulations. This behavior could be related to the low proportions of SA incorporated, which apparently were insufficient to appreciably alter the formation and morphology of the chia oil oleogel microcapsules.

The EE of the three microcapsule formulations ranged from 68.1 ± 1.76 to 71.3 ± 1.25%. Although slight differences in EE values were observed among the formulations, they were not statistically significant (*p* > 0.05), indicating that the partial replacement of GA with SA did not substantially affect oil retention capacity under the conditions evaluated. Consistent with this behavior, incorporating SA at concentrations up to 1.0% (*w*/*w*) enabled the microcapsules to maintain physicochemical and morphological properties comparable to those of the optimal formulation based exclusively on GA. Therefore, the variations observed among the formulations were insufficient to demonstrate a clear effect of SA incorporation on the physicochemical and morphological properties of the chia oil oleogel microcapsules.

#### 2.2.2. Effect of Wall Material Composition on In Vitro Digestion

**Figure 5:**
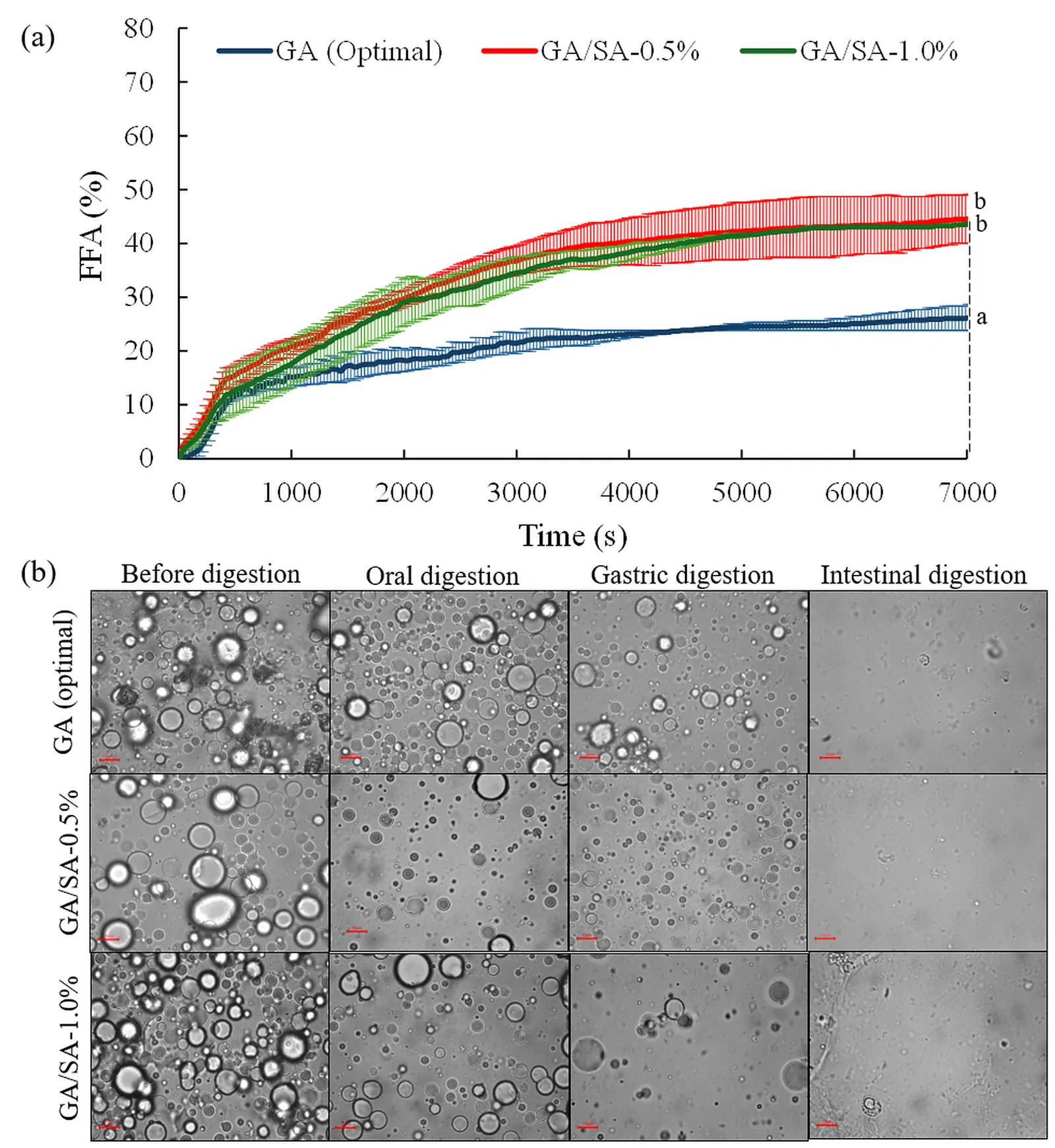
(**a**) Free fatty acid (FFA) release during static in vitro gastrointestinal digestion and (**b**) optical microscopy images (100×) of chia oil oleogel microcapsules formulated with GA (optimal formulation) and different GA/SA ratios, maintaining a constant total wall material content of 15% (*w*/*w*), before digestion and during the oral, gastric, and intestinal phases. Statistical comparisons were performed for the final values at the end of the intestinal phase; different letters indicate significant differences between microcapsules (*p* < 0.05).

The effect of the GA-based wall material composition on the lipid digestion/FFA release of chia oil oleogel microcapsules was evaluated. Additionally, the optimal formulation was compared with formulations containing a GA/SA mixture, while maintaining a constant total wall material content. The resulting chia oil oleogel microcapsules were subjected to static in vitro gastrointestinal digestion, and the extent of lipid hydrolysis was assessed by quantifying the free fatty acids (FFA) released during the intestinal phase (Figure 5).

Figure 5a shows the free fatty acid (FFA) release profile during the in vitro gastrointestinal digestion of the chia oil oleogel microcapsules. The FFA values were calculated from the blank-corrected NaOH consumption, using microcapsules without oil as the digestion blank to account for background alkali consumption from the wall matrix and other non-lipid components. The GA-based formulation showed a progressive increase in FFA release, reaching 26.4 ± 3.23% at the end of the intestinal phase. In contrast, the GA/SA–0.5% and GA/SA–1.0% formulations exhibited higher FFA release, reaching 44.8 ± 6.33% and 43.4 ± 0.30%, respectively. These results indicate that incorporating SA significantly increased (*p* < 0.05) lipid hydrolysis during simulated gastrointestinal digestion, leading to greater release of free fatty acids at the end of the intestinal phase. Notably, this difference occurred despite the absence of significant differences (*p* > 0.05) in moisture content, solubility, and EE among the formulations (Table 1). Because the formulations showed comparable moisture content, dispersibility, EE, and initial morphology, the higher FFA release observed in the SA-containing microcapsules may be attributable to differences in the wall matrix’s response under gastrointestinal conditions. SA is known to exhibit pH- and ion-dependent structural behavior, which may influence matrix hydration and reorganization during digestion and, consequently, the accessibility of bile salts and digestive enzymes to the encapsulated lipid phase [25,26]. However, matrix swelling, disintegration, and enzyme–interface interactions were not directly measured in the present study. Therefore, this explanation should be regarded as a plausible interpretation rather than a demonstrated causal mechanism. Previous studies have likewise shown that the structure of spray-dried microcapsules can influence lipid accessibility by modifying the interactions between bile salts and lipase and the encapsulated oil [22].

This result is also consistent with the microscopic observations in Figure 5b, which show progressive structural changes throughout gastrointestinal digestion, particularly during the intestinal phase, in agreement with the higher lipid hydrolysis observed in Figure 5a. The microscopic observations in Figure 5b provided complementary qualitative evidence of progressive structural changes throughout gastrointestinal digestion, particularly during the intestinal phase. These observations are consistent with the distinct digestive responses shown in Figure 5a but do not, by themselves, establish the mechanism underlying the differences in lipid hydrolysis. Zhang et al. [23] similarly reported partial particle disruption during the gastric phase and residual or coalesced oil droplets during the intestinal phase of spray-dried oil powders. In addition, Singh et al. [25] demonstrated that an alginate coating in a Pickering system underwent marked structural changes in response to gastrointestinal pH, with dissolution at near-neutral pH leading to emulsion release. They also reported a more sustained FFA release than with free oil, which they attributed to changes in the accessibility of bile salts and lipase to the interface during digestion.

The differences in FFA release were not accompanied by evident morphological differences among the microcapsules before digestion, as all formulations exhibited predominantly spherical microcapsules, without evident agglomeration, with particle sizes appearing to be predominantly below 50 μm based on the SEM scale bars (Figure 4). These results suggest that partial replacement of GA with SA altered the microcapsules’ digestive response, increasing lipid hydrolysis compared with the GA formulation. However, no additional increase in final FFA release was observed when the SA content increased from 0.5 to 1.0% (*w*/*w*).

## 3. Conclusions

This study demonstrated the feasibility of producing powdered chia oil oleogel microcapsules by combining wax-structured oleogels, lupin protein aggregate-stabilized Pickering emulsions, and spray drying. Increasing the wax concentration to 5% (*w*/*w*) enhanced oil-binding capacity and firmness, while 3% (*w*/*w*) lupin protein aggregates provided adequate physical stability to the OLG/W Pickering emulsions. Within the evaluated experimental domain, the Taguchi design identified an inlet air temperature of 160 °C, a BW-structured chia oil oleogel, and GA as the wall material as the optimal conditions, yielding an experimental EE of 71.3 ± 1.25%. Although the optimized formulation showed slightly higher EE than the control containing unstructured chia oil, the difference was insufficient to conclusively establish an effect of oleogelation on lipid retention during spray drying. Likewise, the higher EE was not accompanied by greater oxidative stability under the conditions evaluated. Partial replacement of GA with up to 1% (*w*/*w*) SA maintained comparable moisture content, solubility, process yield, EE, and particle morphology, but markedly increased lipid digestion under simulated gastrointestinal conditions, with final FFA release increasing from 26.4 ± 3.23% for GA to 44.8 ± 6.33% and 43.4 ± 0.30% for GA/SA–0.5% and GA/SA–1.0%, respectively. These findings indicate that wall material composition can modulate lipid digestion under simulated gastrointestinal conditions without substantially altering the microcapsules’ initial physicochemical and morphological characteristics. The system provides a promising platform for investigating how wall material composition can be used to modify the digestive behavior of powdered chia oil oleogel ingredients.

## 4. Materials and Methods

### 4.1. Materials

Shellac wax (SW), SSB^®^ Cera 2, was acquired from SSB Stroever GmbH & Co. KG. (Bremen, Germany). Beeswax (BW), gum arabic (GA), maltodextrin (MD) and sodium alginate (SA) were purchased from Sigma Aldrich (St. Louis, MO, USA). Chia oil was purchased from Aceitería Dumont (Santiago, Chile). Lupin protein isolate from Lupinus luteus (95% of protein content in dry basis, <5% of moisture) (Burgos-Díaz et al. [32]) was provided by CGNA. All other chemicals used were of analytical grade.

### 4.2. Preparation of Chia Oil Oleogels

Chia oil oleogels were prepared using SW and BW, according to the methodology described by Morales et al. [29]. The oleogelation process was carried out on a hot plate at 82 °C for 30 min, using oleogelant concentrations of 2.5 and 5.0% (*w*/*w*) and constant stirring at 300 rpm. The molten chia oil oleogels were then allowed to cool at room temperature for at least 2 h, after which they were stored in the dark at 4 °C. Samples were analyzed after 24 h.

#### 4.2.1. Oil Binding Capacity (OBC)

OBC was measured according to Millao et al. [8]. Briefly, 1 g of oleogel was placed in a 15 mL tube and centrifuged at 7000 rpm for 40 min. The tube was then inverted to remove the released oil, and the remaining sample was weighed. OBC was calculated using Equation (1).

(1) $$OBC\left(\%\right)=1-\frac{m_{i}-m_{f}}{m_{i}}\times 100$$

where *m_i_* and *m_f_* represent the sample mass before centrifugation and after oil drainage, respectively.

#### 4.2.2. Texture Analysis

Firmness was determined as a textural property of the chia oil oleogels, according to the procedure described by Millao et al. [8]. For the measurement, 30 g of each oleogel sample was transferred into a glass container and stored at 4 °C for 24 h. The samples were removed from storage immediately before testing and subjected to a penetration assay using a TA.XT PlusC texture analyzer (Stable Micro Systems, Surrey, UK) equipped with a 5 kg load cell. A 5 mm cylindrical probe was employed to penetrate the sample inside the container to a depth of 10 mm at a constant speed of 1 mm/s. The probe was then returned to its initial position at the same speed. The maximum positive force recorded during penetration was taken as the sample’s firmness and expressed in grams.

### 4.3. Chia Oil Oleogel-in-Water (OLG/W) Pickering Emulsions

#### 4.3.1. Preparation of Lupin Protein Aggregates as Pickering Particles

The Pickering particle dispersions were prepared following the methodology described by Burgos-Díaz et al. [31]. Dry lupin protein isolate was dispersed in distilled water at concentrations of 1, 2, 3 and 4% (*w*/*w*), and the pH was adjusted to 7 using 1 M NaOH or HCl. The solutions were stirred for 1 h at room temperature and then stored overnight at 4 °C to ensure complete hydration. After hydration, the dispersions were centrifuged at 5000 rpm for 5 min to remove the precipitate. The supernatants were then heated to 80 °C for 30 min and rapidly cooled to form the lupin protein aggregates (LPA).

#### 4.3.2. Preparation of OLG/W Pickering Emulsions

OLG/W Pickering emulsions were prepared by mixing 90% (*w*/*w*) of the LPA dispersion with 10% (*w*/*w*) of the 5.0% (*w*/*w*) wax-structured chia oil oleogel. The oil phase consisted of chia oil structured with either shellac wax (SW) or beeswax (BW), as described in Section 4.2. The oleogels were melted at 80 °C and mixed with the LPA dispersions, which were maintained at the same temperature. The resulting mixtures were homogenized at 10,000 rpm for 5 min using a high-speed homogenizer. LPA concentrations of 1, 2, 3, and 4% (*w*/*w*) were evaluated in the aqueous phase.

#### 4.3.3. Creaming Index of OLG/W Pickering Emulsions

Creaming stability was assessed by tracking changes in the creaming index (CI) over a 24 h period, according to the procedure reported by Burgos-Díaz et al. [39]. For this analysis, 5 mL of each Pickering emulsion were transferred to glass tubes, which were subsequently sealed to minimize water evaporation. The CI was expressed as a percentage (%) and calculated using Equation (2):

(2) $$CI(\%)=\left(H_{s}|/|H_{e}\right)\times 100$$

where *H_s_* corresponds to the serum layer height, and *H_e_* is the total emulsion height.

#### 4.3.4. Apparent Viscosity of OLG/W Pickering Emulsions

Apparent viscosity was measured at 25 °C using a VISCO™ rotational viscometer (ATAGO^®^,Tokyo, Japan). The A2L configuration, comprising the A2 spindle and l-type beaker, was operated at 250 rpm.

#### 4.3.5. Microstructure of OLG/W Pickering Emulsions and Powdered OLG Microcapsules

The microstructure of the OLG/W Pickering emulsions and powdered OLG microcapsules was analyzed by optical microscopy (Euromex, Diuve, The Netherlands). Microcapsules were examined before and after in vitro digestion to assess changes in their microstructure, following the methodology described by Zhang et al. [22]. A small portion of each sample was positioned in the center of a microscope slide and carefully covered with a glass coverslip. Images were acquired at room temperature and subsequently processed and analyzed using ImageJ software (version 1.45s, National Institutes of Health, USA), including the addition of scale bars. For the OLG/W Pickering emulsions, the equivalent diameter (Deq) was calculated according to Ferreira and Rasband [40] using Equation (3):

(3) $$D_{eq}=2\times \sqrt{\frac{area}{\pi }}$$

The phase distribution of the structured chia oil in the OLG/W Pickering emulsions was analyzed using an Olympus FV1000 spectral confocal microscope (Olympus Corporation, Tokyo, Japan). Nile red (30 μM in acetone) was used to stain the oil phase, with excitation/emission wavelengths set at 488/590 nm, respectively. Rhodamine B was used to stain the lupin protein aggregates, with excitation at 543 nm and emission at 625 nm. The acquired images were visualized using FV-ASW software (v. 1.7).

The morphology of the powdered OLG oleogel microcapsules was observed by scanning electron microscopy (SEM) using a JEOL JSM-6380 LV microscope (JEOL Ltd., 3-1-2 Musashino, Akishima, Tokyo 196-8558, Japan).

### 4.4. Spray Drying of OLG/W Pickering Emulsions

Spray drying of the O/W and OLG/W emulsions was performed using a B-290 Mini Spray Dryer (Büchi, Flawil, Switzerland), following the method described by Morales et al. [41]. Before spray drying, the O/W and OLG/W emulsions were mixed with the encapsulating wall material (WM), which was added as a dry powder, to obtain a final spray-drying feed consisting of 85% (*w*/*w*) emulsion and 15% (*w*/*w*) total WM. Thus, the final formulation was 100% (*w*/*w*). The 15% (*w*/*w*) WM concentration was selected based on practical requirements for spray-drying feed preparation and on previous reports on the use of carbohydrate-based wall materials for oil- and food-ingredient microencapsulation by spray drying [22,41]. The total WM concentration was kept constant across all formulations to allow the effect of WM type to be evaluated independently of wall-material concentration. The mixtures were stirred at 400 rpm for 2 h to ensure complete incorporation of the WM before spray drying. The resulting mixtures were then spray-dried at a feed rate of 8 mL/min and an aspiration rate of 85%. The resulting microcapsules were collected and stored in sealed plastic bags, protected from light, and refrigerated at 4 °C until further characterization.

### 4.5. Experimental Design

To optimize the formulation of chia oil oleogel microcapsules, a Taguchi experimental design using an L_4_ (2^3^) orthogonal array was employed. Three independent variables, each evaluated at two levels, were investigated using the “higher-is-better” optimization criterion, with the oil phase encapsulation efficiency (EE) as the response variable. The independent variables were inlet air temperature (120 and 160 °C), emulsion type (OLG_SW_/W–E and OLG_BW_/W–E) and WM type (MD and GA). Additionally, the accelerated oxidative stability of all formulations obtained from the experimental design was evaluated by measuring the induction period (IP). The powders obtained were collected and stored in the dark at 4 °C until analysis.

Rao et al. [42] reported that traditional optimization evaluates one variable at a time, which can involve a large number of experiments and require substantial time, cost, and labor. In contrast, the Taguchi design uses orthogonal arrays to efficiently estimate main effects with a minimum number of experimental runs, thereby determining the contributions of the design variables and their optimal combination. In the present study, the predictive performance of the Taguchi method was evaluated using Minitab 19, based on the average response for each factor level. The optimized theoretical equation (OTE) was obtained by averaging the responses with the greatest impact, allowing the identification of the most relevant variables and their working levels for microcapsule EE. The OTE was calculated using Equation (4):

(4) $$OTE=T+\left[{WL}_{Temperature}-T\right]+\left[{WL}_{Emulsion\;type}-T\right]+\left[{WL}_{WM\;type}-T\right]$$

where *T* represents the overall average response obtained from the experimental runs, while WL denotes the working level of each variable included in the equation.

Table 2 presents the orthogonal array employed in the experimental design, together with the corresponding levels of the design variables.

**Table 2:** Encapsulation efficiency (EE) using the orthogonal matrix L_4_ (2^3^).

Design Point	Temperature (°C)	Emulsion Type	WM Type	EE (%)
1	120	OLG_SW_/W–E	MD	55.3 ± 1.06
2	120	OLG_BW_/W–E	GA	68.0 ± 0.71
3	160	OLG_SW/_W–E	GA	70.5 ± 1.41
4	160	OLG_BW_/W–E	MD	65.8 ± 1.06

Following the identification of GA as the optimal wall material in the Taguchi experimental design, a second formulation stage was conducted using the same optimal spray-drying conditions. GA was partially replaced with sodium alginate (SA) at 0.5 and 1.0% (*w*/*w*), while maintaining the total wall material concentration at 15% (*w*/*w*). The wall materials were incorporated into the previously selected Pickering emulsion as a dry powder mixture. Accordingly, the formulations containing 0.5 and 1.0% (*w*/*w*) SA consisted of 14.5 and 14.0% (*w*/*w*) GA, respectively, together with 85% (*w*/*w*) Pickering emulsion, giving a total formulation of 100% (*w*/*w*). These formulations were subsequently spray-dried at the optimal inlet air temperature identified by the experimental design, and the resulting microcapsules were evaluated to assess the effect of SA incorporation on in vitro gastrointestinal digestion.

### 4.6. Characterization of Chia Oil Oleogel Microcapsules

#### 4.6.1. Oil Phase Encapsulation Efficiency (EE)

The EE of chia oil oleogel microcapsules was evaluated following the method of Zhang et al. [22], with some modifications, by measuring the total and surface oil contents. To determine the total oil content, 2 g of microencapsulated powder were transferred to a Whatman No. 1 cellulose extraction thimble (Cytiva, Whatman™, Marlborough, MA, USA), and lipids were extracted with 150 mL of petroleum ether for 5 h in a Soxhlet apparatus. After extraction, the solvent was evaporated, and the extracted lipids were dried at 70 °C until a constant weight was obtained. For surface oil determination, 2 g of microencapsulated powder was dispersed in 30 mL of petroleum ether and stirred gently at 50 rpm for 1 min at room temperature. The resulting suspension was filtered, after which the solvent was removed with a rotary evaporator. The residual lipid fraction was then dried at 105 °C to constant weight. The EE was calculated according to Equation (5):

(5) $$EE(\%)=\left[\left(Total\;oil\;phase-Surface\;oil\;phase\right)|/|Total\;oil\;phase\right]\times 100$$

#### 4.6.2. Yield of the Spray-Drying Process

Spray-drying yield was determined following the procedure reported by Morales et al. [34]. The yield was expressed as the percentage of recovered powder (W_2_) relative to the total solids (W_1_) introduced into the spray dryer with the OLG/W Pickering emulsion after incorporation of the wall material (WM). All determinations were carried out in triplicate, and the yield was calculated according to Equation (6):

(6) $$Yield\;(\%)=W_{2}/W_{1}\times 100$$

#### 4.6.3. Moisture Content

The moisture content of the powdered chia oil oleogel microcapsules was determined according to AOAC method 934.06. Briefly, 2 g of the powdered sample was dried in a forced-air oven (BOV-T50F, Fremont, CA, USA) at 105 °C for 2 h. After drying, the samples were cooled to room temperature in a desiccator and weighed using an analytical balance. Moisture content was determined in triplicate and calculated according to Equation (7):

(7) $$M(\%)=\left(M_{1}-M_{2}\;\right)/\left(M_{1}-M_{0}\right)\times 100$$

where *M_0_* is the mass of the empty crucible, *M_1_* is the mass of the crucible containing the sample before drying, and M_2_ is the mass of the crucible containing the dried sample.

#### 4.6.4. Powder Solubility

Powder solubility was determined following the gravimetric procedure reported by Morales et al. [34] with some modifications. For this purpose, 0.5 g of powdered chia oil oleogel microcapsules was suspended in 50 mL of distilled water and stirred at 100 rpm for 30 min at room temperature using an orbital shaker. The suspension was then centrifuged at 3500 rpm for 5 min, after which a 25 mL aliquot of the clear supernatant was carefully collected and transferred to a pre-weighed porcelain dish. The sample was dried at 105 °C for 2 h until constant weight, and the solubility was calculated in triplicate according to Equation (8):

(8) $$Solubility\;(\%)=\left(M_{2}\times 2\right)/\left(M_{1}\right)\times 100$$

where *M_2_* is the final mass in grams and *M_1_* is the initial mass in grams.

### 4.7. Accelerated Oxidative Stability

The accelerated oxidative stability of the microcapsules obtained from the experimental design, including the optimized formulation and the unstructured chia oil control, was evaluated using an OXITEST reactor (VELP Scientifica, Milan, Italy), following the procedure described by Chóez-Guaranda et al. [37] with some modifications. For the analysis, a 5 g portion of each sample was weighed and uniformly placed in the cells. The instrument temperature and oxygen pressure were adjusted to 90 °C and 6 bar, respectively. Oxisoft ^TM^ 3.0.0 software(VELP Scientifica, Usmate (MB), Italy) was used to determine the oxidative stability of the samples, which was reported as the induction period (IP).

### 4.8. In Vitro Gastrointestinal Digestion

Powdered chia oil oleogel microcapsules formulated with 15% (*w*/*w*) of the optimized wall material and supplemented with different concentrations of sodium alginate (SA) (0, 0.5, and 1.0%, *w*/*w*) were subjected to a static in vitro gastrointestinal digestion protocol based on INFOGEST 2.0 [43], with the modifications described below. The digestion protocol comprised oral, gastric, and intestinal phases. Simulated salivary (SSF), gastric (SGF), and intestinal (SIF) fluids were prepared according to the INFOGEST protocol and pre-equilibrated at 37 °C before digestion. The microcapsules were dispersed in distilled water to obtain a food matrix containing a 4% (*w*/*w*) oil phase.

During the oral phase, 5 g of sample was mixed with SSF, CaCl_2_ (0.3 M), and distilled water, followed by incubation at 37 °C for 2 min under continuous rotation (15 rpm). The resulting oral bolus (10 mL) was transferred to the gastric phase, where it was combined with SGF, CaCl_2_ (0.3 M), and freshly prepared pepsin solution. The pH was adjusted to 3.0 using 1 M HCl; the final volume was brought to 20 mL with distilled water, and the mixture was incubated at 37 °C for 2 h under continuous rotation (15 rpm).

For the intestinal phase, the gastric chyme was mixed with SIF, CaCl_2_ (0.3 M), pancreatin solution (100 U/mL trypsin activity), bile salts (10 mM), and pancreatic lipase (2000 U/mL activity). Distilled water was added to obtain a final volume of 40 mL, and the pH was maintained at 7.0 by automatic titration with 0.1 M NaOH at 37 °C for 2 h using a pH-stat titrator.

The extent of lipid digestion was determined from the percentage of free fatty acids (FFA) released during the intestinal phase of the simulated gastrointestinal digestion using the pH-stat method described by Ciuffarin et al. [44]. The volume of 0.1 M NaOH consumed during intestinal digestion was continuously recorded. A digestion blank consisting of microcapsules without oil was subjected to the same gastrointestinal digestion procedure and used to account for background NaOH consumption associated with the wall matrix and other non-lipid components. The corrected NaOH volume was calculated as:

$$V_{NaOH,corr}=V_{NaOH,sample}-V_{NaOH,black}$$

and used to determine the extent of lipid digestion according to Equation (9):

(9) $$FFA(\%)=100\times \left(\frac{V_{NaOH,corr}\times {10^{-3}\times MW}_{oil}\times C_{NAOH}}{m_{oil}\times 2}\right)$$

*V_NaOH, corr_* is the blank-corrected volume of NaOH (mL) required to neutralize the released FFA, *C_NaOH_* is the NaOH concentration (mol/L), *m*_oil_ is the mass of chia oil in the reaction vessel (g), and MW_oil_ is the average molecular weight of chia oil (885.432 g mol^−1^). The calculation assumes that pancreatic lipase hydrolyzes two fatty acids per triglyceride molecule.

### 4.9. Statistical Analysis

Unless otherwise indicated, all experiments were carried out in triplicate, and the data are presented as mean ± standard deviation. Treatment effects were statistically assessed using one-way analysis of variance (ANOVA) with Tukey’s multiple comparison test at *p* < 0.05. Statistical analyses were conducted using Minitab 19 software (Minitab LLC, State College, PA, USA).

## Figures and Tables

**Table 1:** Characterization of chia oil oleogel microcapsules obtained by spray drying.

Microcapsules	Moisture (%)	Solubility (%)	Yield (%)	EE (%)
GA (Optimal)	4.08 ± 0.16	73.2 ± 1.90	37.9 ± 1.44	71.3 ± 1.25
GA/SA–0.5%	4.36 ± 0.10	76.7 ± 2.12	35.2 ± 1.38	70.4 ± 2.96
GA/SA–1.0%	4.71 ± 0.23	76.6 ± 1.66	36.4 ± 3.04	68.1 ± 1.76

## Data Availability

The original contributions presented in this study are included in the article/Supplementary Materials. Further inquiries can be directed to the corresponding author.

## Supplementary Materials

The following supporting information can be downloaded at: https://www.mdpi.com/article/10.3390/gels12090856/s1, Figure S1: Optical microscopy images of 3% (*w*/*v*) lupin protein dispersion before and after thermal treatment (80 °C for 30 min) followed by cooling. (a) Untreated lupin protein dispersion (control); (b) thermally treated and cooled dispersion showing the formation of visible protein aggregates; Figure S2: Visual appearance of the control O/W Pickering emulsion prepared with unstructured chia oil during storage; Figure S3: Size distribution, expressed as equivalent diameter, of (a) OLG_SW_/W-E and (b) OLG_BW_/W-E stabilized with 3% (*w*/*w*) LPA.; Table S1: Accelerated oxidative stability of the samples from the experimental design, the optimal formulation, and the control prepared with unstructured chia oil, expressed as induction period (IP).; Table S2: Apparent viscosity of OLG/W Pickering emulsions containing GA and GA/SA mixtures as wall materials prior to spray drying.

## References

[1] Orsavova J., Misurcova L., Ambrozova J.V., Vicha R., Mlcek J. (2015). Fatty acids composition of vegetable oils and its contribution to dietary energy intake and dependence of cardiovascular mortality on dietary intake of fatty acids. Int. J. Mol. Sci..

[2] Ixtaina M.L., Martínez V., Spotorno C.M., Mateo D.M., Maestri B.W.K. (2011). Characterization of chia seed oils obtained by pressing and solvent extraction. J. Food Compos. Anal..

[3] Saini R.K., Prasad P., Sreedhar R.V., Akhilender Naidu K., Shang X., Keum Y.-S. (2021). Omega−3 Polyunsaturated Fatty Acids (PUFAs): Emerging Plant and Microbial Sources, Oxidative Stability, Bioavailability, and Health Benefits—A Review. Antioxidants.

[4] Hashemi B., Varidi M., Assadpour E., Zhang F., Jafari S.M. (2024). Natural Oleogelators for the Formulation of Oleogels by Considering Their Rheological and Textural Perspective; a Review. Int. J. Biol. Macromol..

[5] Naeli M.H., Milani J.M., Farmani J., Zargaraan A. (2022). Developing and Optimizing Low-Saturated Oleogel Shortening Based on Ethyl Cellulose and Hydroxypropyl Methyl Cellulose Biopolymers. Food Chem..

[6] Han W., Chai X., Liu Y., Xu Y., Tan C.-P. (2022). Crystal Network Structure and Stability of Beeswax-Based Oleogels with Different Polyunsaturated Fatty Acid Oils. Food Chem..

[7] Winkler-Moser J.K., Hwang H.-S., Felker F.C., Byars J., Peterson S. (2023). Increasing the Firmness of Wax-Based Oleogels Using Ternary Mixtures of Sunflower Wax with Beeswax:Candelilla Wax Combinations. J. Am. Oil Chem. Soc..

[8] Millao S., Quilaqueo M., Contardo I., Rubilar M. (2025). Enhancing the Oxidative Stability of Beeswax–Canola Oleogels: Effects of Ascorbic Acid and Alpha-Tocopherol on Their Physical and Chemical Properties. Gels.

[9] Hwang H., Fhaner M., Winkler-Moser J.K., Liu S.X. (2018). Oxidation of Fish Oil Oleogels Formed by Natural Waxes in Comparison With Bulk Oil. Eur. J. Lipid Sci. Technol..

[10] Patel A. (2018). Chapter 7—Shellac-Based Oleogels. Edible Oleogels.

[11] Yuan Y., He N., Xue Q., Guo Q., Dong L., Haruna M.H., Zhang X., Li B., Li L. (2021). Shellac: A Promising Natural Polymer in the Food Industry. Trends Food Sci. Technol..

[12] Patel A., Dewettinck K. (2016). Edible oil structuring: An overview and recent updates. Food Funct..

[13] Wei F., Miao J., Tan H., Feng R., Zheng Q., Cao Y., Lan Y. (2021). Oleogel-structured emulsion for enhanced oxidative stability of perilla oil: Influence of crystal morphology and cooling temperature. LWT Food Sci. Technol..

[14] Qi W., Li T., Zhang Z., Wu T. (2021). Preparation and characterization of oleogel-in-water pickering emulsions stabilized by cellulose nanocrystals. Food Hydrocoll..

[15] Rajaei A., Nahalkar A. (2026). Gum Arabic–based Pickering emulsions: Design principles and emerging applications. Carbohydr. Polym..

[16] Udoetok I.A., Mohamed M.H., Wilson L.D. (2024). Stabilization of Oil-in-Water Pickering Emulsions by Surface-Functionalized Cellulose Hydrogel. Gels.

[17] Davtalab M., Naji-Tabasi S., Shahidi-Noghabi M., Martins A.J., Bourbon A.I., Cerqueira M.A. (2024). Pickering Emulsion Stabilized by Different Concentrations of Whey Protein–Cress Seed Gum Nanoparticles. Foods.

[18] Yu J., Yun M., Li J., Gao Y., Mao L. (2024). Development of Oleogel-in-Water High Internal Phase Emulsions with Improved Physicochemical Stability and Their Application in Mayonnaise. Foods.

[19] Genc E., Karasu S., Akcicek A., Toker O.-S. (2024). Fabrication and characterisation of Pickering emulsion-based oleogel stabilised by citrus fibre and whey protein isolate colloidal complex: Application in cookie formulation. Int. J. Food Sci. Technol..

[20] Gharsallaoui A., Roudaut G., Chambin O., Voilley A., Saurel R. (2007). Applications of spray-drying in microencapsulation of food ingredients: An overview. Food Res. Int..

[21] Mohammed N.K., Tan C.P., Manap Y.A., Muhialdin B.J., Hussin A.S.M. (2020). Spray drying for the encapsulation of oils—A review. Molecules.

[22] Zhang X., Li L., Li J., Liang H., Chen Y., Li B., Xiaogang Luo X., Pei Y., Liu S. (2022). Edible oil powders based on spray-dried Pickering emulsion stabilized by soy protein/cellulose nanofibrils. LWT Food Sci. Technol..

[23] Carneiro H.C.F., Tonon R.V., Grosso C.R.F., Hubinger M.D. (2013). Encapsulation efficiency and oxidative stability of flaxseed oil microencapsulated by spray drying using different combinations of wall materials. J. Food Eng..

[24] Chang C.C., Varankovich N., Nickerson M.T. (2016). Microencapsulation of canola oil by lentil protein isolate-based wall materials. Food Chem..

[25] Singh C.K.S., Lim H.-P., Tey B.-T., Chan E.-S. (2021). Spray-dried alginate-coated Pickering emulsion stabilized by chitosan for improved oxidative stability and in vitro release profile. Carbohydr. Polym..

[26] Alvarez R., Robert R., Quintriqueo A., Oyarzún-Ampuero F., Mackie A., Torcello-Gómez A. (2024). Characterization and in vitro bioaccessability of optimized chia oil-Capsul-sodium alginate microparticles obtained by 3 nozzle spray-drying. Food Bioprod. Process..

[27] Blake A.I., Co E.D., Marangoni A.G. (2014). Structure and Physical Properties of Plant Wax Crystal Networks and Their Relationship to Oil Binding Capacity. J. Am. Oil Chem. Soc..

[28] Doan C.D., Tavernier I., Okuro P.K., Dewettinck K. (2018). Internal and External Factors Affecting the Crystallization, Gelation and Applicability of Wax-Based Oleogels in Food Industry. Innov. Food Sci. Emerg. Technol..

[29] Morales E., Marilaf K., Rubilar M., Contardo I., Quilaqueo M., Millao S., Bustamante M., Burgos-Díaz C., Garrido-Miranda K. (2025). Rheological and Physicochemical Characterization of Structured Chia Oil: A Novel Approach Using a Low-Content Shellac Wax/Beeswax Blend as Oleogelant. Gels.

[30] Gao Y., Li M., Zhang L., Wang Z., Yu Q., Han L. (2021). Preparation of Rapeseed Oil Oleogels Based on Beeswax and Its Application in Beef Heart Patties to Replace Animal Fat. LWT Food Sci. Technol..

[31] Burgos-Díaz C., Opazo-Navarrete M., Soto-Añual M., Leal-Calderón F., Bustamante M. (2020). Food-grade Pickering emulsion as a novel astaxanthin encapsulation system for making powder-based products: Evaluation of astaxanthin stability during processing, storage, and its bioaccessibility. Food Res. Int..

[32] Burgos-Díaz C., Wandersleben T., Olivos M., Lichtin N., Bustamante M., Solans C. (2019). Food-grade Pickering stabilizers obtained from a protein-rich lupin cultivar (AluProt-CGNA^®^): Chemical characterization and emulsifying properties. Food Hydrocoll..

[33] Wu J., Shi M., Li W., Zhao L., Wang Z., Yan X. (2015). Pickering emulsions stabilized by whey protein nano particles prepared by thermal cross-linking. Colloids Surf. B Biointerfaces.

[34] Morales E., Burgos-Díaz C., Zúñiga R.N., Jorkowski J., Quilaqueo M., Rubilar M. (2021). Influence of O/W emulsion interfacial ionic membranes on the encapsulation efficiency and storage stability of powder microencapsulated astaxanthin. Food Bioprod. Process..

[35] Al-Ismail K.M., Mehya G., Al-Khatib H.S., Al-Dabbas M. (2015). Effect of microencapsulation of cardamom’s essential oil in gum Arabic and whey protein isolate using spray drying on its stability during storage. Qual. Assur. Saf. Crops Foods.

[36] Mangope K., Kaseke T., Fawole O. (2025). Spray-drying microencapsulation of fixed oils: An innovative and sustainable technology to enhance oxidative stability, functionality and application in food systems. Appl. Food Res..

[37] Chóez-Guaranda I., Mosi-Roa Y., Chacón-Fuentes M., Garrido-Miranda K., Opazo-Navarrete M., Coronel-León J., Burgos-Díaz C. (2025). Enhancing the Oxidative Stability of Flaxseed Oil Using Food-Grade O/W Pickering Emulsions Stabilized by Antioxidant-Rich Cocoa Byproduct Particles. Appl. Food Res..

[38] Gimenez P.A., Bergesse A.E., Camacho N., Ribotta P.D., Martínez M.L., González A. (2022). Chia Oil Microencapsulation by Spray Drying Using Modified Soy Protein as Wall Material. Biol. Life Sci..

[39] Burgos-Díaz C., Leal-Calderon F., Mosi-Roa Y., Chacón-Fuentes M., Garrido-Miranda K., Opazo-Navarrete M., Quiroz A., Bustamante M. (2024). Enhancing the Retention and Oxidative Stability of Volatile Flavors: A Novel Approach Utilizing O/W Pickering Emulsions Based on Agri-Food Byproducts and Spray-Drying. Foods.

[40] Ferreira T., Rasband W. (2012). ImageJ User Guide Version IJ 1.46 r. http://imagej.nih.gov/ij/docs/guide.

[41] Morales E., Burgos-Díaz C., Zúñiga R.N., Jorkowski J., Quilaqueo M., Rubilar M. (2021). Effect of interfacial ionic layers on the food-grade O/W emulsion physical stability and astaxanthin retention during spray-drying. Foods.

[42] Rao R.S., Kumar C.G., Prakasham R.S., Hobbs P.J. (2008). The Taguchi methodology as a statistical tool for biotechnological applications: A critical appraisal. Biotechnol. J..

[43] Brodkorb A., Egger L., Alminger M., Alvito P., Assunção R., Ballance S., Bohn T., Bourlieu-Lacanal C., Boutrou R., Carrière F. (2019). INFOGEST Static in Vitro Simulation of Gastrointestinal Food Digestion. Nat. Protoc..

[44] Ciuffarin F., Alongim M., Plazzotta S., Lucci P., Schena P., Manzocco L., Calligaris S. (2023). Oleogelation of extra virgin olive oil by different gelators affects lipid digestion and polyphenol bioaccessibility. Food Res. Int..

