# Supplementary material for: Spray-Dried Chia Oil Oleogel Microcapsules from Pickering Emulsions: Wall Material Effects on Encapsulation Efficiency and In Vitro Lipid Digestion
Source: Gels. 2026 Sep 20;12(9):856. doi: 10.3390/gels12090856 (PMC13606417; doi:10.3390/gels12090856)
Supplement: Supplementary file 1 [file gels-12-00856-s001.zip › gels-4543516-supplementary.pdf]

Supplementary Material

Figures

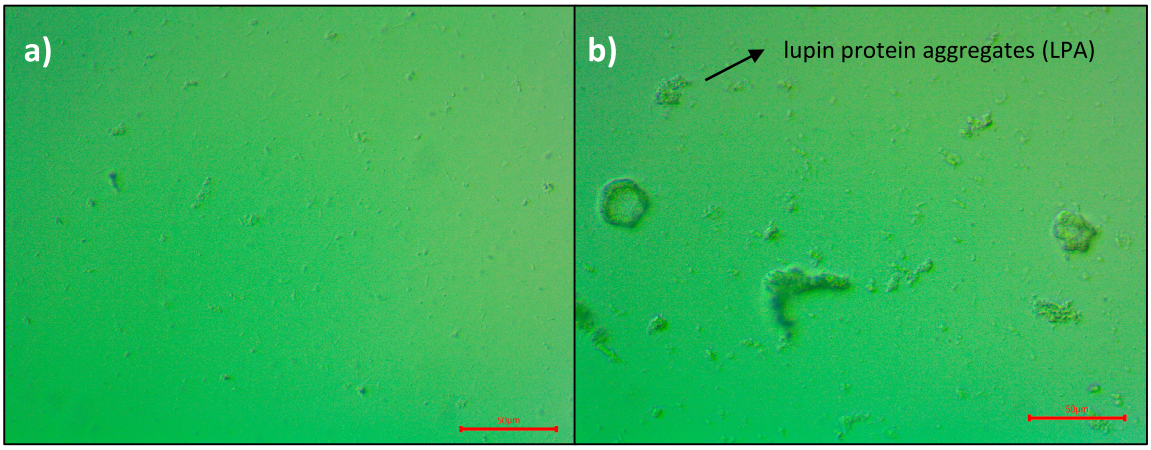

Figure S1. Optical microscopy images of 3% (w/v) lupin protein dispersion before and after thermal treatment (80 °C for 30 min) followed by cooling. (a) Untreated lupin protein dispersion (control); (b) thermally treated and cooled dispersion showing the formation of visible protein aggregates.

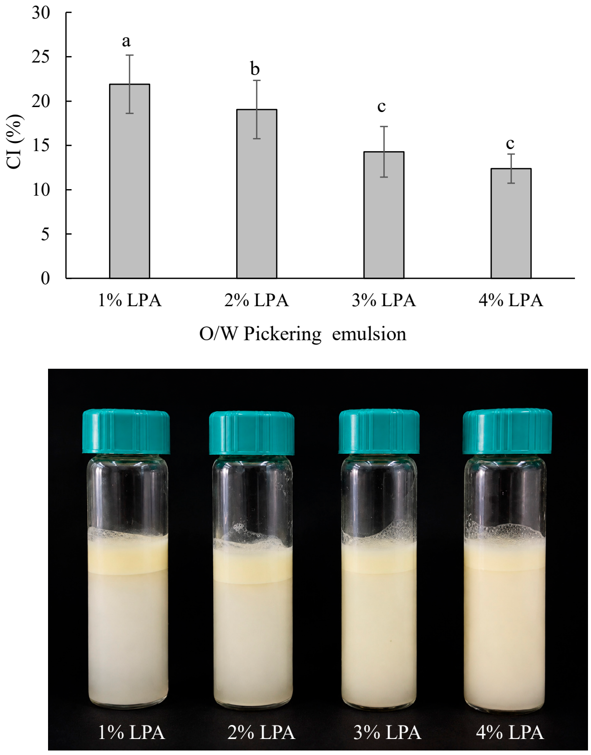

Figure S2. Visual appearance of the control O/W Pickering emulsion prepared with unstructured chia oil during storage.

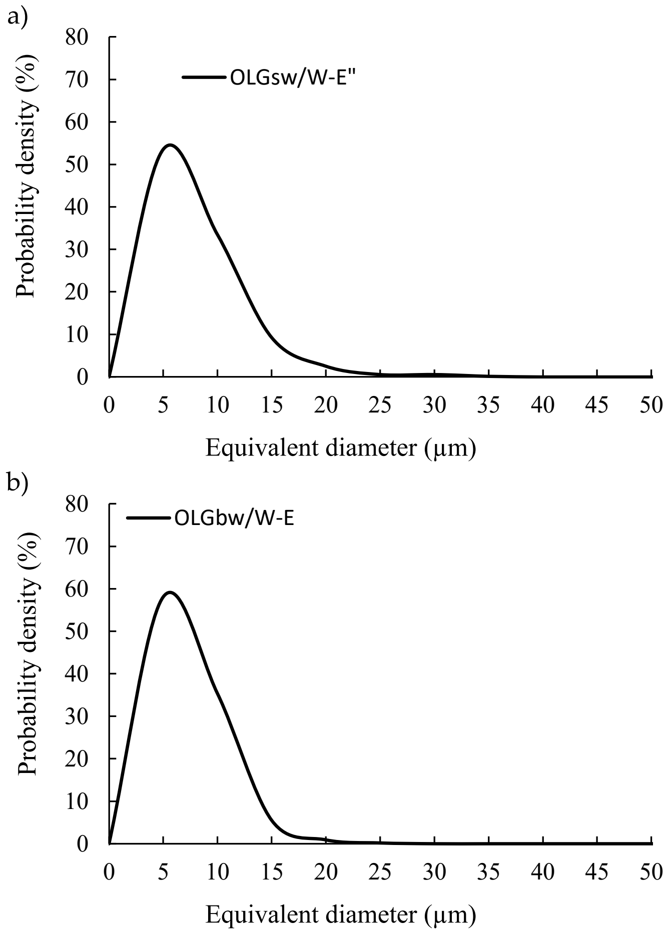

Figure S3. Size distribution, expressed as equivalent diameter, of (a) OLG_{SW}/W-E and (b) OLG_{BW}/W-E stabilized with 3% (w/w) LPA.

Table S1. Accelerated oxidative stability of the samples from the experimental design, the optimal formulation, and the control prepared with unstructured chia oil, expressed as induction period (IP).

Desing point	Temperature (°C)	Emulsion type	WM type	PI (h)
1	120	$OLG_{SW/}$W-E	MD	3.41 ± 0.05
2	120	$OLG_{BW/}$W-E	GA	3.44 ± 0.05
3	160	$OLG_{SW/}$W-E	GA	3.46 ± 0.03
4	160	$OLG_{BW/}$W-E	MD	3.39 ± 0.04
**Optimal**	**160**	**$OLG_{BW/}$W-E**	**GA**	**3.43 ± 0.03**
**Control**	**160**	**O/W-E**	**GA**	**3.44 ± 0.05**

Table S2. Apparent viscosity of OLG/W Pickering emulsions containing GA and GA/SA mixtures as wall materials prior to spray-drying.

OLG/W Pickering emulsions containing WM	Apparent viscosity (Cp)
GA (Optimal)	50 ± 1.41 c
GA/SA-0.5%	61 ± 2.12 c
GA/SA-1.0%	121 ± 2.83 b
GA/SA-1.5%	200 ± 4.24 a
